# Snail-Regulated MiR-375 Inhibits Migration and Invasion of Gastric Cancer Cells by Targeting JAK2

**DOI:** 10.1371/journal.pone.0099516

**Published:** 2014-07-23

**Authors:** Yanjun Xu, Juan Jin, Yiman Liu, Zhenxia Huang, Yujie Deng, Tao You, Tianhua Zhou, Jianmin Si, Wei Zhuo

**Affiliations:** 1 Zhejiang Cancer Hospital, Hangzhou, China; 2 Department of Cell Biology and Program in Molecular cell Biology, Zhejiang University School of Medicine, Hangzhou, China; 3 Department of Surgery, 2nd Affiliated Hospital of Wenzhou Medical College, Wenzhou, China; 4 Institute of Gastroenterology, Zhejiang University, Hangzhou, China; 5 Sir Run Run Shaw Hospital, Zhejiang University School of Medicine, Hangzhou, China; University of South Alabama, United States of America

## Abstract

MicroRNAs (miRNAs) have been reported to play a critical role in cancer invasion and metastasis. Our previous study showed that miR-375 frequently downregulated in gastric cancer suppresses cell proliferation by targeting Janus kinase 2 (JAK2). Here, we further found that the expression level of miR-375 is significantly decreased in metastatic gastric cancer tissues compared with the non-metastasis controls. Ectopic expression of miR-375 inhibits the migration and invasion of gastric cancer cells partially by targeting JAK2. Furthermore, miR-375 expression is negatively regulated by the metastasis associated transcription factor Snail, which directly binds to the putative promoter of miR-375. Moreover, overexpression of Snail can partially reverse the inhibition of gastric cancer cell migration caused by miR-375. Taken together, these data suggest that miR-375 may be negatively regulated by Snail and involved in gastric cancer cell migration and invasion potentially by targeting JAK2.

## Introduction

Metastasis is the most terrible aspects of cancer and has been studied for more than 100 years [1], [2]. In gastric cancer, the high mortality mainly attributes to delayed diagnosis because of the lack of specific symptoms in early stage. And metastasis is responsible for the gastric cancer-related mortality [3], [4]. Migration and invasion of cancer cells are essential processes during cancer metastatic procession which consists of a series of interrelated steps, including proliferation, detachment, circulation, transport, arrest in organs, adherence to vessel wall, extravasation, establishment of a microenvironment, and proliferation in distant organs. In gastric cancer, cells invasion into the surrounding tissue is a crucial early step [3], [5]. However, the mechanisms of gastric cancer cells migration, invasion and metastasis have not been fully understood.

In recent years, various molecules, for instance, growth factors, cytokines, extracellular matrix-remodeling molecules, and some transcription factors such as Snail, Twist and ZEB1 [6], [7], [8], [9], [10], [11], have been revealed to drive the progress of cancer cells migration, invasion and metastasis. Lately, it has become evident that, in addition to abnormalities in protein-coding genes, alterations in non-coding genes can also contribute to the cancer cells migration, invasion and metastasis, such as miRNAs, which are a class of small single-stranded non-coding RNA molecules that regulate gene expression with great potential and have been implicated in the regulation of cancer cells migration, invasion and metastasis as activators or suppressors [12], [13], [14], [15], [16]. To date, a number of miRNAs have been studied to be implicated in gastric cancer metastasis progression, for example, miR-218, miR-9, miR-7, and miR-146a [6], [17], [18], [19]. We have studied the association between specific dysregulated miRNA and specific metastasis step of gastric cancer, which will provide insights into the potential mechanisms of gastric cancer cells migration, invasion and metastasis.

In our previous study, miR-375 was significantly downregulated in gastric cancer and inhibited gastric cancer cells proliferation by targeting JAK2 [20]. Interestingly, in the present study, we further found that the expression level of miR-375 was even lower in gastric cancer samples from metastasis-positive patients compaired with that from metastasis-free patients. Thus, we proposed that miR-375 might have a causal role in gastric cancer metastasis. Our studies uncovered that ectopic expression of miR-375 inhibited the migration and invasion of gastric cancer cells also partially by targeting JAK2. We further prompted to find out how miR-375 expression was regulated in gastric cancer. Results indicated that miR-375 was a target of the metastasis associated transcription factor Snail and its expression was inversely correlated with Snail in gastric cancer. Overexpression of Snail can partially reverse the inhibition of gastric cancer cell migration caused by miR-375. Thus, our findings demonstrate that miR-375 inhibits gastric cancer cells migration and invasion through Snail/miR-375/JAK2 regulation pathway.

## Materials and Methods

### Clinical samples (Ethics Statement) and cell lines

Clinical gastric cancer specimens and their pair-matched non-malignant gastric samples from 39 patients undergoing gastric cancer resection were provided by Sir Run Run Shaw Hospital (Hangzhou, China). All the samples were collected with written consent from the patients as described previously [20]. Both gastric tumor tissues and adjacent nontumorous gastric tissues collected after surgery were and divided into two parts. One was frozen in liquid nitrogen immediately for further use, another part was stored in formalin for pathology analysis. The patients involved in our study were separated into metastasis-free and metastasis-positive groups (9/30). The gastric cancer cell lines (AGS and MGC-803) and one non-malignant gastric epithelial cell line (GES-1) were described previously [20].

### RNA extraction

Total RNA from gastric samples and cell lines was extracted using the mirVana miRNA Isolation Kit (Ambion, TX, USA) following the manufacture's protocol.

### Quantitative real-time PCR analysis

The expression of miR-375 was assayed using the Taqman MicroRNA Assays (Applied Biosystems, CA, USA) with specific primers (P/N: 4373151, Applied Biosystems). Reverse transcription reaction was done starting from 10 ng of total RNA using the looped primers. Quantitative real-time PCR (qRT-PCR) was performed using the standard Taqman MicroRNA Assays protocol on ABI7500 Real-Time PCR Detection System. The ΔΔCt method for relative quantization was used to determine miRNA expression. The Ct is the fractional cycle number at which the fluorescence of each sample passes the fixed threshold. The ΔCt was calculated by subtracting the Ct of snRNA U6 (RNU6B, P/N: 4373381, Applied Biosystems) from the Ct of the miRNA of interest. The ΔΔCt was calculated by subtracting the ΔCt of the reference sample (paired non-malignant tissue for surgical samples, normal tissues and GES-1 cell for gastric cancer cell lines) from the ΔCt of each sample. Fold change was determined as 2^−ΔΔCt^.

### Migration and invasion assay

Cells were transfected with 20 nM pre-miR-375 or negative control using Transfection Agent (Ambion, TX, USA) following manufacture's protocol in 24-well plates. 24 h after transfection, Transwell migration assay and Matrigel invasion assay were performed separately using 24-well Transwell inserts with 8 µm pore size (Corning Costar Corp). For Transwell migration assay, 2×10^4^ AGS or 3×10^4^ MGC-803 cells suspended in 100 µl corresponding culture medium without fetal bovine serum (FBS) were loaded into the top chamber of transwell insert with non-coated membrane. For Matrigel invasion assay, 5×10^4^ AGS or MGC-803 cells were plated in 100 µl serum-free medium in the upper Matrigel-coated chamber instead. In both assays, the bottom chamber was containing 600 µl medium with 20% FBS. Cells were then allowed to migrated or invaded for 12 h at 37 °C. The cells that migrated or invaded into the bottom chamber were fixed, stained with 4′, 6-diamidino-2-phenylindole (DAPI, 1∶1000), visualized under phase contrast microscope and photographed. Total number of migrated or invaded cells was counted by IPP (Image-Pro Plus 6.0) software. All experiments were independently repeated at least three times.

### Scratch-wound healing assay

Cells were transfected as previously described and allowed to grow to confluence. The cells were then cultured in corresponding medium without FBS for 12 h and then scratched with a pipette tip. Wound areas were marked and photographed at 0 h, 12 h, 24 h and 36 h respectively. The rate of cells migration was evaluated by both photographing and quantifying the migrated distance of cells moved from the wound edge toward the center using IPP 6.0 system. All experiments were repeated three times.

### Constructs

To produce pGL3-375pro plasmid, the DNA sequence containing pri-miR-375 (primary miR-375) promoter was amplified by RT-PCR using the primers 5′-ATCG CTCGAG ACA GAC CCT GCT AAG CGA CTC-3′ and 5′-ATCG AAGCTT ACG CCT TGG AGC TTG TCC-3′ and then cloned into pGL3-basic vector (Promega). To construct the plasmid that expresses Snail in cells, the open reading frame (ORF) sequence of Snail was cloned into mammalian expression vector pEGFP-C1 (Clontech, CA, USA) by using the primers 5′- ATCG AA GCT TCG ATG CCG CGC TCT TTC CTC G-3′ and 5′-ATCG GGA TCC TCA GCG GGG ACA TCC TGA GCA-3′. For ectopic expression of FLAG-tagged JAK2, human JAK2 with coding region was cloned into pCMV-Tag 2C vector. To construct the plasmid that expresses miR-375 in mammalian cells, the paired oligonucleotides based on the primary sequence of has-miR-375 and its flanking regions were cloned into mammalian expression vector pEGFP-C1 (Clontech, CA, USA). All of the constructs were confirmed by sequencing.

### Luciferase assay

Forty thousand cells were seeded in 24-well plates 24 h prior to transfection. Cells were transfected with either pGL3-375pro or pGL3-Basic vector. The pRL-TK vector (Promega, WI, USA) containing *Renilla* luciferase was also cotransfected as a reference control. Firefly and *Renilla* luciferase activities were measured by using Dual-Luciferase Reporter Assay (Promega) 24 h after transfection. Firefly luciferase activity was normalized to Renilla luciferase activity.

### Statistical analyses

Data are represented as mean ± standard error (SE) of three independent experiments. Relationships between the expression of miR-375 and the expression of Snail mRNA were explored by *Pearson's* correlation coefficient, which was described previously [20]. Student's *t*-test and *X* ^2^ test were performed to determine statistical significance. *P*<0.05 was considered to be statistically significant.

## Results

### MiR-375 is dramatically downregulated in gastric cancer cells and tissues from metastasis-positive patients

**Figure 1:**
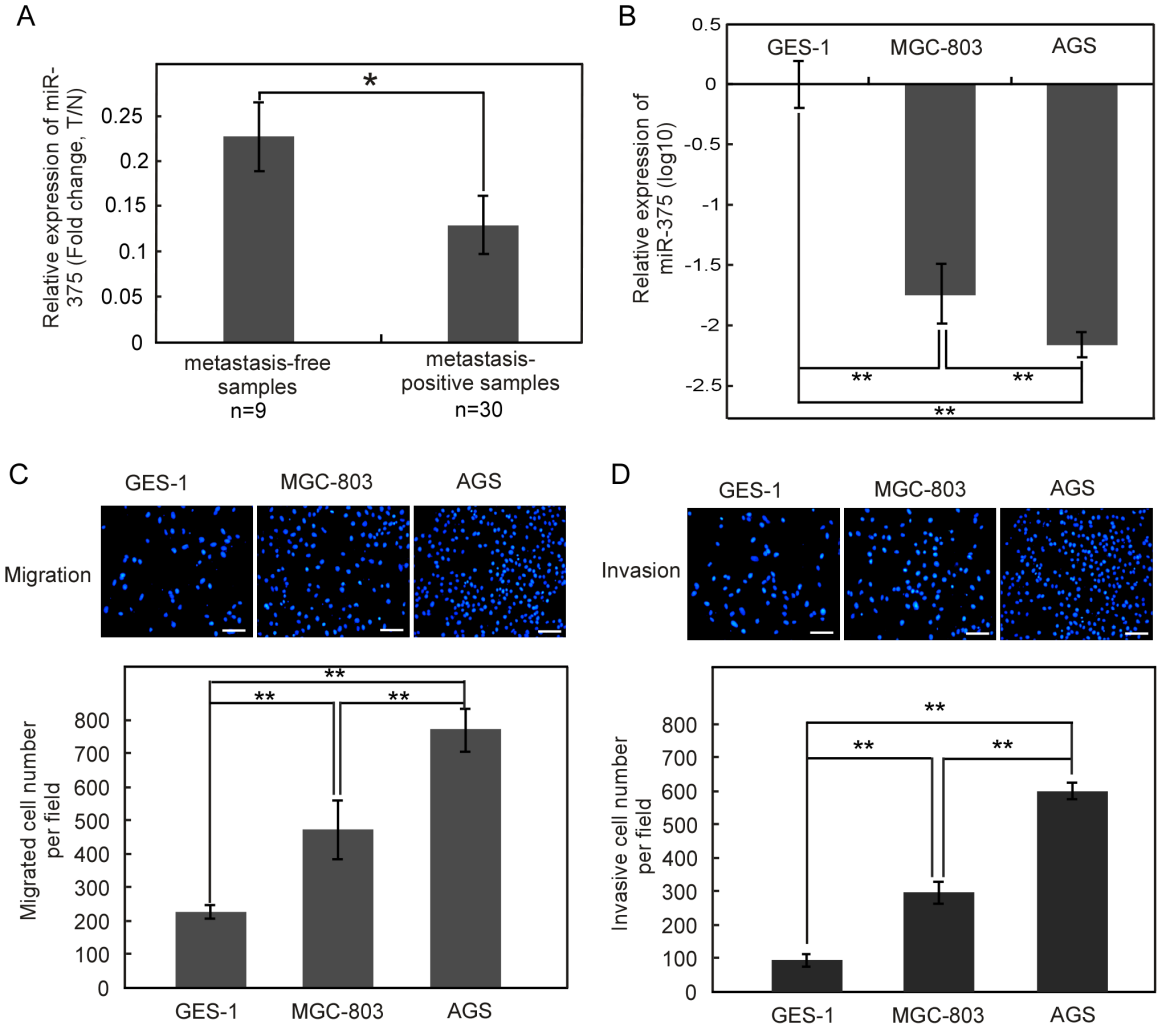
MiR-375 is downregulated in samples from metastasis-positive patients and cells with greater migration and invasion abilities. The expression of miR-375 was investigated by qRT-PCR. (A) Relative fold changes (Tumor tissue/Normal tissue, T/N) between gastric cancer samples from metastasis-free or –positive patients and their adjacent non-malignant tissues. The expression level of miR-375 was nearly two-fold reduction in tissues from 30 metastasis-positive patients than that from 9 metastasis-free patients, *P<0.05. (B) The expression level of miR-375 in three human gastric epithelial cell lines with different migration and invasion abilities. The expression level of miR-375 in AGS cells was lower than that of MGC-803 and GES-1 cells. **P<0.01. (C, D) The migration and invasion abilities of the three gastric epithelial cell lines were measured with transwell chambers. Photos are representative fields of migrated (C) or invasive (D) cells on the membrane. Bar graphs represent the average number of cells on the underside of membrane ± SE. **P<0.01 as compared with non-malignant gastric epithelial cell line GES-1.

To figure out whether miR-375 is associated with gastric cancer metastasis, we first detected the expression level of miR-375 in primary gastric cancer tissues from metastasis-positive and metastasis-free patients. When compared with samples from metastasis-free patients, the expression level of miR-375 was nearly two-fold reduction in tissues from metastasis-positive patients (*P*<0.05) (**Figure 1A**). In consonance with this result, the expression level of miR-375 in gastric cell lines was negatively associated with the abilities of cells migration and invasion. The metastatic properties of gastric epithelial cell lines (GES-1, MGC-803, AGS) were characterized. As shown in **Figure 1C and 1D**, migration and invasion abilities of AGS cell line were greater than that of MGC-803 and GES-1 cell lines (*P*<0.01). Inversely, miR-375 expression in AGS cells was lower than that of MGC-803 and GES-1 cells (*P*<0.01) (**Figure 1B**). In a word, miR-375 is downregulated in gastric cancer tissues from metastasis-positive patients and in gastric cancer cells with greater migration and invasion abilities. This correlation indicates that miR-375 might have a causal role in gastric cancer metastasis.

### Overexpression of miR-375 inhibits gastric cancer cells migration and invasion

**Figure 2:**
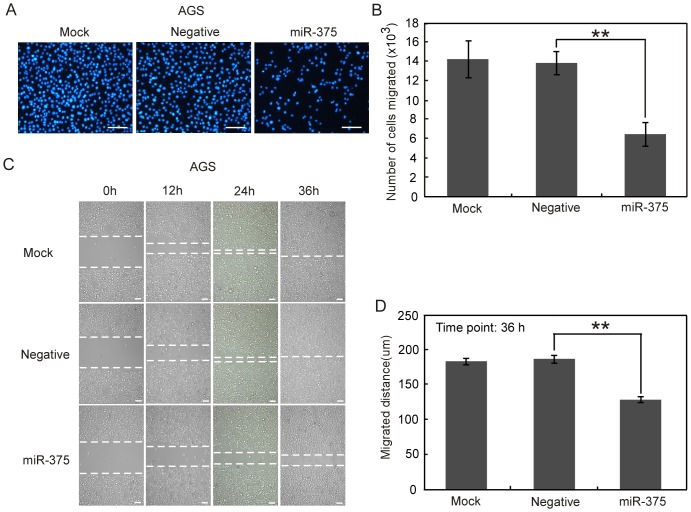
Ectopic expression of miR-375 suppresses the migration of AGS cells. AGS cells transfected with miR-375 precursor (miR-375), negative control (Negative) or neither of the above (Mock) were subjected to Transwell migration assay (A) and scratch-wound healing analysis (C). (A) Representative fields of migrated cells on the underside of membrane which were fixed and stained with DAPI. (B) Total number of migrated cells on the underside of membrane was counted by IPP 6.0 software. (C) The cells migration to the wounded area was photographed by microscopy at 0 h, 12 h, 24 h and 36 h post-wounding. The dotted lines indicate the areas lacking cells. (D) The rate of migration was examined by measuring the distance of cells moved from the wound edge toward the center in 36 h after scratching. The data are presented as mean ± SE of at least three independent experiments. Bars, 50 µm. **P<0.01.

**Figure 3:**
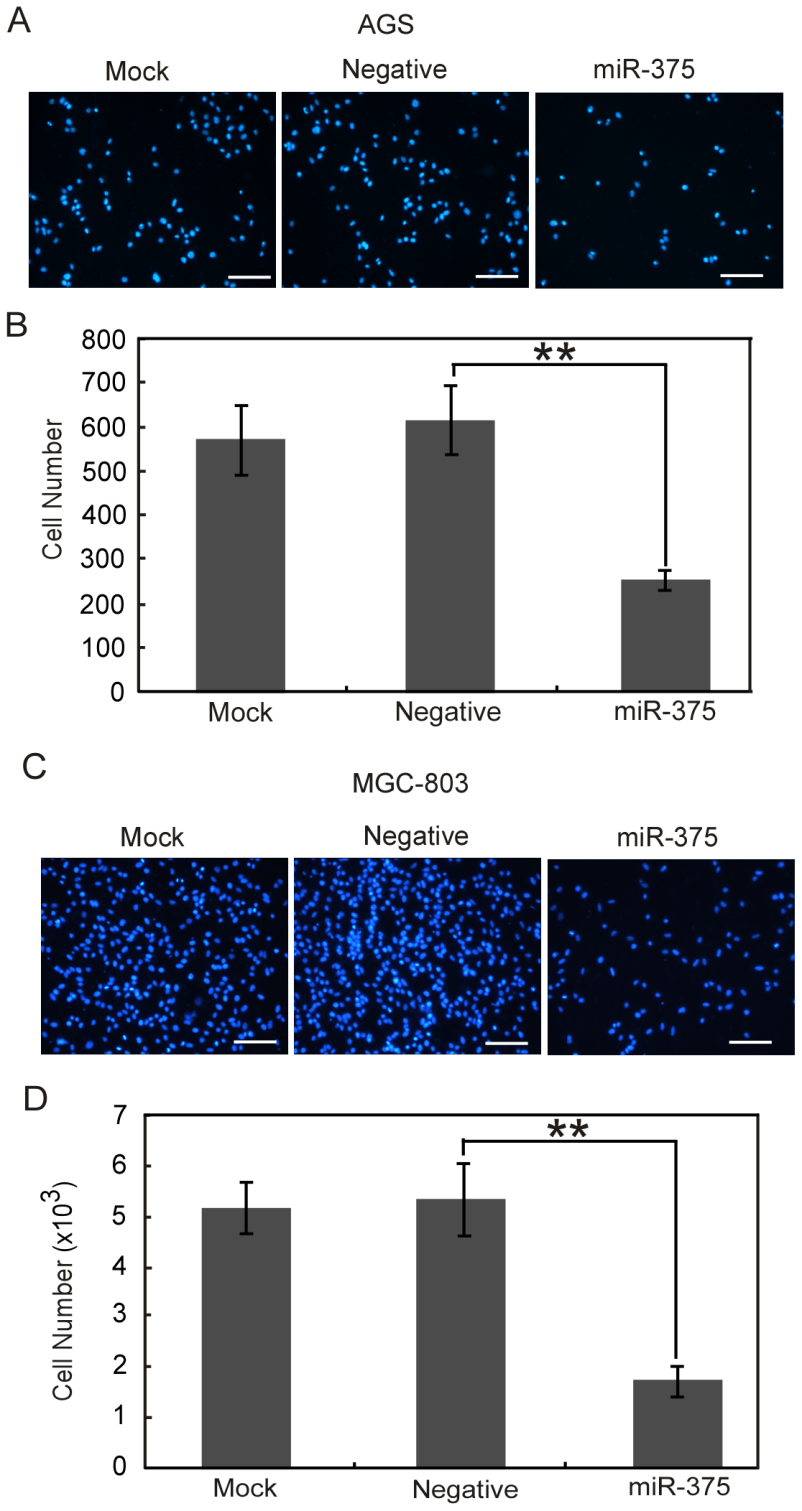
Ectopic expression of miR-375 suppresses the invasion of gastric cancer cells. AGS and MGC-803 cells transfected with pre-miR-375 (miR-375), negative control (Negative) or neither of the above (Mock) were subject to Matrigel invasion analysis. (A, C) Representative fields of the cells on the bottom chamber at 12 h post invasion were shown. Scale bars, 50 µm. (B, D) The total number of invasive cells on the underside of membrane was counted by IPP 6.0. Data are shown as mean ± SE from three independent experiments. **P<0.01.

To explore the role of miR-375 in gastric cancer metastasis, we examined the effect of miR-375 overexpression on the migration and invasion of AGS and MGC-803 gastric cancer cells with low endougenous expression of miR-375. Cells were transfected with either miR-375 precursor (miR-375) or precursor-negative control oligonucleotides (Negative) or neither of the above (Mock). The Transwell migration assay showed that overexpression of miR-375 greatly inhibited the migration of AGS (**Figure 2A, 2B**) and MGC-803 cells (**Figure S1A, S1B**). In consistent with these results, the scratch-wound healing assay also revealed that the velocities of AGS (**Figure 2C, 2D**) and MGC-803 cells (**Figure S1C, S1D**) migration toward the wound area were significantly reduced after overexpression of miR-375. We further employed Matrigel invasion assay and found that overexpression of miR-375 led to a more than two-fold reduction in the invasive properties of AGS (**Figure 3A, 3B**) and MGC-803 cells (**Figure 3C, 3D**). Collectively, these results indicate that overexpression of miR-375 is sufficient to inhibit both the migration and invasion abilities of gastric cancer cells.

### MiR-375-regulated JAK2 is involved in the regulation of gastric cancer cells migration and invasion

**Figure 4:**
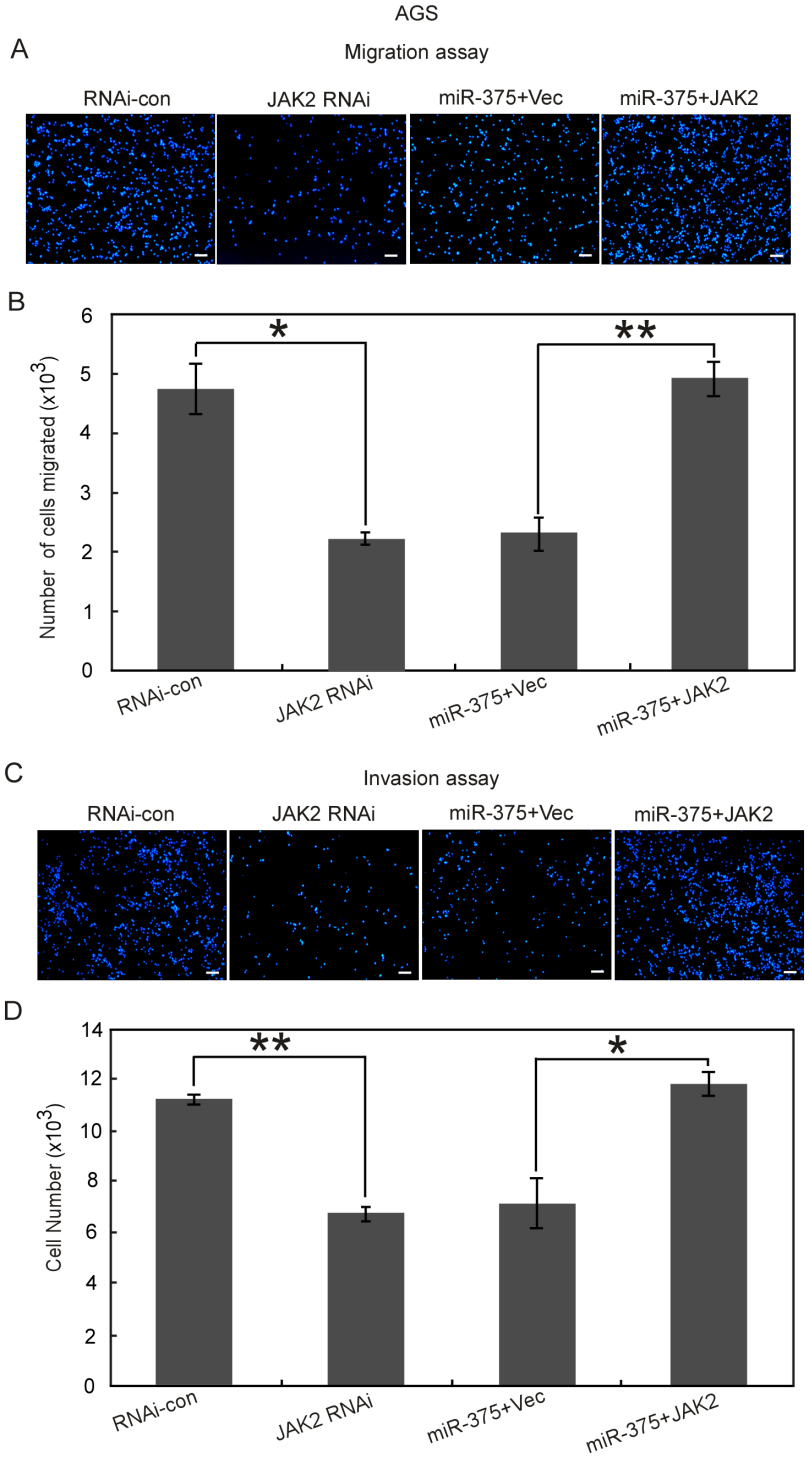
Overexpression of JAK2 reverses miR-375 induced inhibition of gastric cancer cells migration and invasion. The cells transfected with the indicated vectors or oligonucleotides were subjected with Transwell migration (A) or Matrigel invasion assay (C). The rescue experiments for miR-375 overexpression were performed by ectopic expression of JAK2 without 3'-UTR in miR-375-treated cells. (A, C) Representative fields of the cells on the bottom chamber at 12 h post migration or invasion were shown. Scale bars, 50 µm. (B, D) The total number of migrated or invasive cells from nine randomly chosen fields was counted by IPP 6.0. *P<0.05, **P<0.01.

Our previous study has identified JAK2 as a downstream target of miR-375 [20]. Here, we are interested in studying whether JAK2 is involved in the regulation of gastric cancer cells migration and invasion. We employed vector-based RNAi technique to deplete endogenous JAK2 and found that the migration and invasion activities of AGS (**Figure 4**) and MGC-803 cells (**Figure S2**) were both inhibited greatly. Simultaneously, we studied whether JAK2 could counteract the inhibition effect of gastric cancer cells migration and invasion caused by miR-375. Cells were co-transfected with miR-375 precursor and either JAK2 overexpression vector (miR-375+JAK2) or control empty vector (miR-375+Vec). Overexpression of JAK2 clearly promoted cells migration and invasion as shown in **Figure 4** and **S2**. Thus, in consistent with our hypothesis, miR-375 may inhibit the migration and invasion of gastric cancer cells partially by targeting JAK2. We further determined that JAK2 overexpression has no effect on the expression level of miR-375 as shown in **Figure S3**. However, we cannot exclude the possibility that the dysregulation of miR-375 interferes with other targets required for gastric cancer cells migration and invasion.

### Snail downregulates miR-375 expression

**Figure 5:**
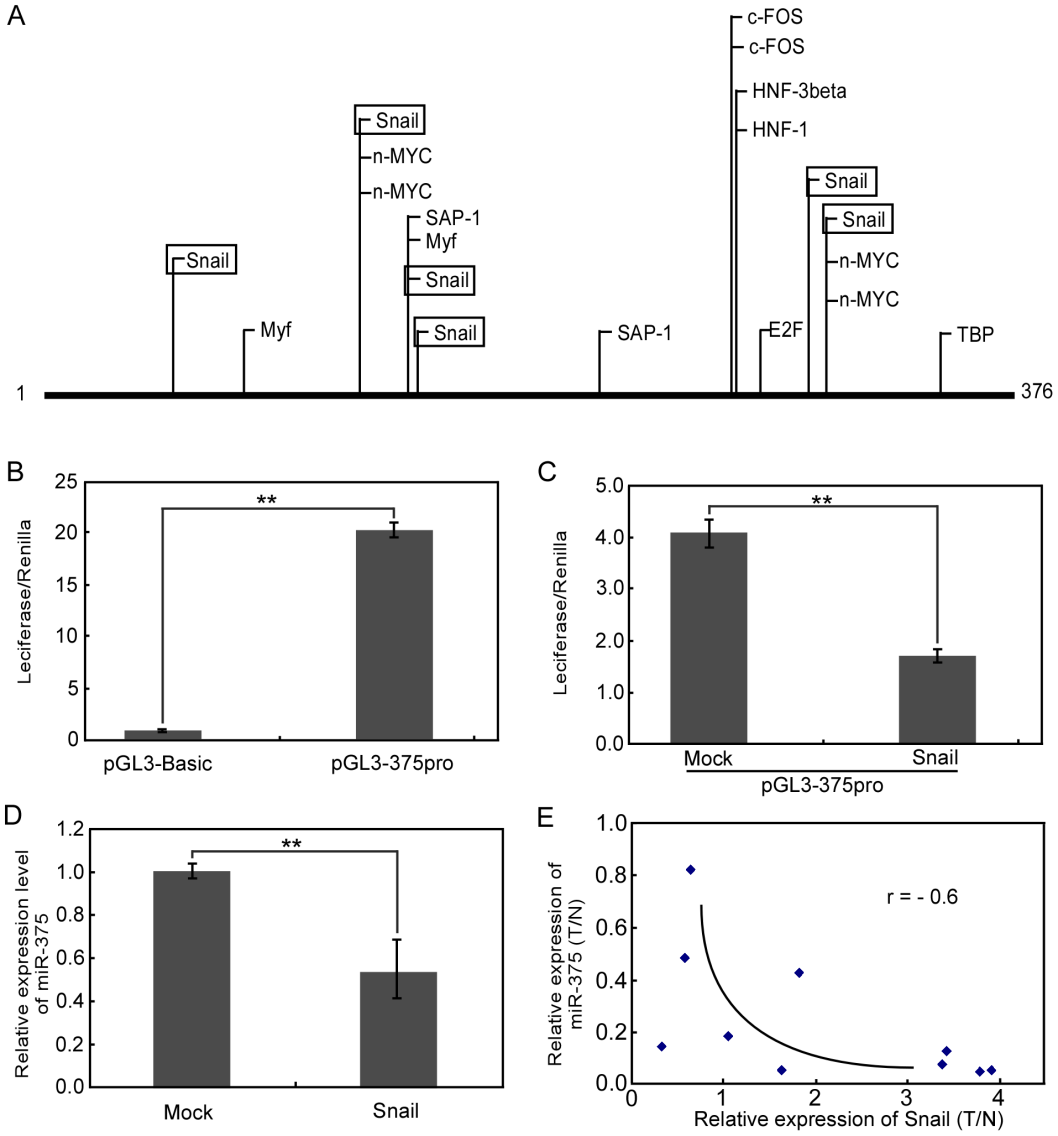
MiR-375 is regulated by Snail. (A) Bioinformatics analysis using the Consite program revealed potential binding sites and found that there were six binding sites of transcription factor Snail in the DNA region which containing the pri-miR-375 gene promoter. (B) The putative promoter region of the miR-375 gene was ligated upstream to the firefly luciferase reporter gene in the promoter-less pGL3-basic vector (pGL3-375pro), resulting in a 20-fold increase in the luciferase activity. (C) Luciferase activity of pGL3-375pro in cells transduced by Snail vector. Luciferase activity was efficiently repressed when pGL3-375pro vector was co-transfected with Snail expression vector. (D) The expression level of miR-375 in cells transduced by Snail overexpressing vector. Snail overexpression could reduce the expression level of miR-375 by 46%. (E) Inverse correlation between miR-375 expression and Snail mRNA level in primary gastric cancer tissues. A statistically significant correlation between miR-375 and Snail was observed by *Pearson's* method with a correlation coefficient of -0.6. **P<0.01.

The above results indicate that miR-375 may be targeted by certain transcription factors that are associated with cancer invasion and metastasis process. Naturally, we focus on how miR-375 expression is regulated. We first found out a conversed DNA region upstream of pri-miR-375 gene, which was reported to contain the pri-miR-375 gene promoter [21]. We then performed Consite program (http://asp.ii.uib.no:8090/cgi-bin/CONSITE/consite) and found that there were six binding sites of transcription factor Snail in the DNA region (**Figure 5A**). The DNA region was cloned into pGL3-basic vector (Promega) (pGL3-375pro) to measure the promoter activity. In consistent with the data from Walker group [21], our results showed that this DNA region contains the pri-miR-375 gene promoter and is capable of directing luciferase expression (**Figure 5B**). Luciferase activity was efficiently repressed when pGL3-375pro vector was co-transfected with Snail expression vector (Snail) but not when co-transfected with the empty control vector (Mock) (**Figure 5C**). Moreover, Snail overexpression could reduce the expression level of miR-375 by 46% (**Figure 5D**). In order to determine the clinical relevance of these results, we further correlated the expression level of miR-375 with the Snail mRNA level in the same gastric cancer patients. As shown in **Figure 5E**, a distinct inverse correlation was found between the expression level of miR-375 and Snail mRNA (*P*<0.05, r = −0.6). Hence, these observations demonstrate that Snail is a potential upstream regulator of miR-375 expression.

### Snail is involved in the regulation of gastric cancer cells migration by targeting miR-375

**Figure 6:**
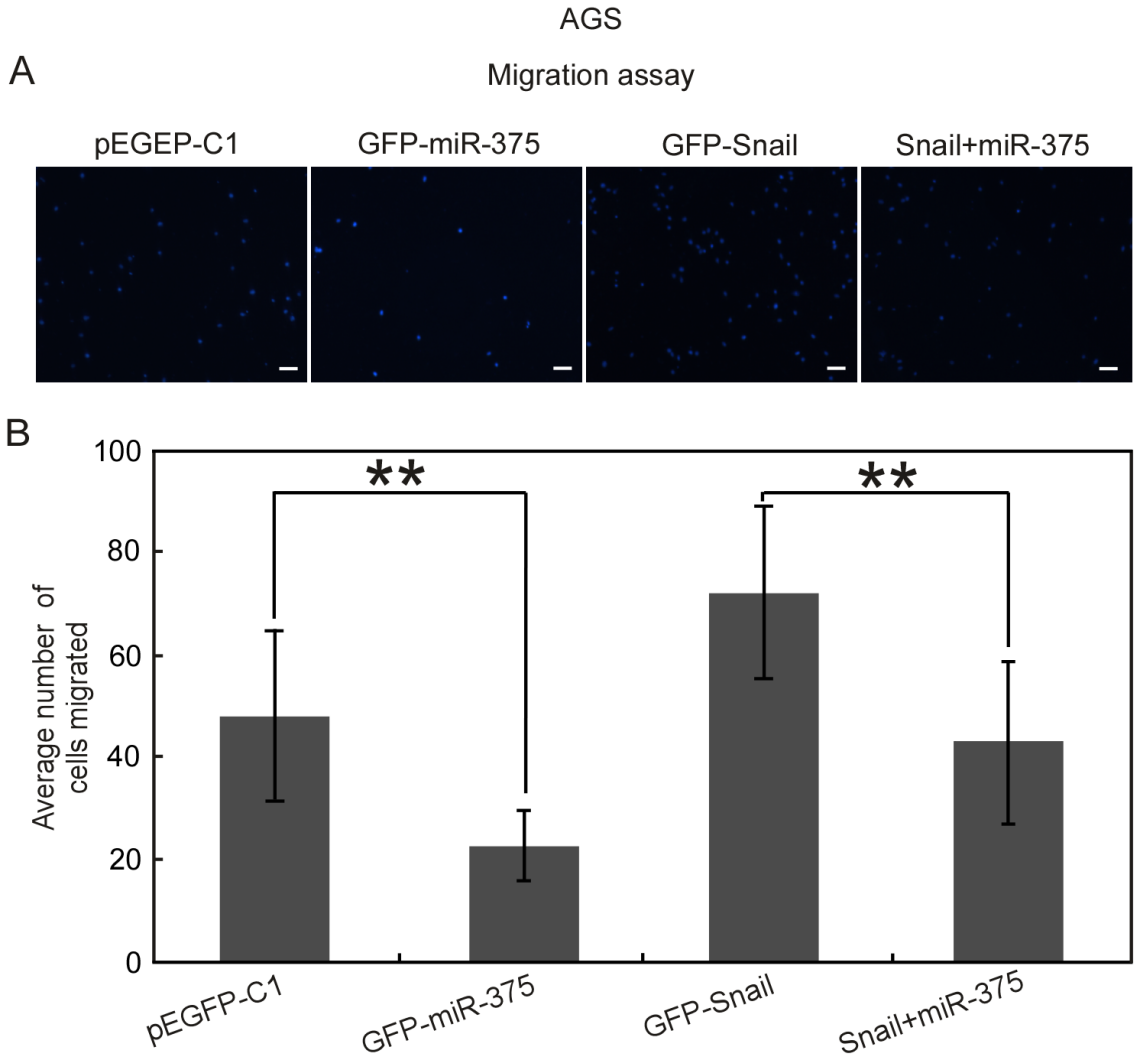
Overexpression of Snail reverses miR-375 induced inhibition of gastric cancer cells migration. The cells transfected with the indicated vectors were subjected with Transwell migration assay. (A) Representative fields of the cells on the bottom chamber at 12 h post migration were shown. Scale bars, 50 µm. (B) The average number of migrated cells from nine randomly chosen fields was counted by IPP 6.0. **P<0.01.

To further elucidate the correlation of miR-375 and Snail, we studied whether Snail is involved in the regulation of gastric cancer cells migration by targeting miR-375. We employed vector-based technique to upregulate Snail expression level and found that the migration activity of AGS cells (**Figure 6**) were promoted greatly. Simultaneously, we studied whether Snail could counteract the inhibition effect of gastric cancer cells migration caused by miR-375. Cells were co-transfected with miR-375 overexpression vector and Snail overexpression vector (Snail + miR-375). Overexpression of Snail clearly promoted cells migration and could partially counteract the inhibition effect of gastric cancer cells migration caused by miR-375 as shown in **Figure 6**. Thus, in consistent with our hypothesis, Snail may inhibit the migration of gastric cancer cells partially by targeting miR-375.

## Discussion

MiRNAs have been reported to regulate tumor migration, invasion and metastasis including gastric cancer [6], [22]. MiR-375 was previously demonstrated to play an important role in gastric cancer cells proliferation [20], [23]. However, the regulatory mechanisms remain unclear. In the present study, we further explored the function and potential mechanisms of miR-375 in the migration and invasion of gastric cancer cells. We found that overexpression of miR-375 inhibited proliferation, migration, and invasion of gastric cancer cells partially by targeting JAK2. It has been over a decade since JAK2 was first cloned [24]. JAK2 is expressed in nearly every tissue and associated with many pathologic progresses. Although it is evident that JAK2 acts as an oncogene in both myeloproliferative disorders and some solid tumors [25], [26], [27], no direct involvement of JAK2 in cancer migration, invasion or metastasis has been reported. Excitingly, our study first demonstrated that knocking down JAK2 by RNAi inhibited the migration and invasion activities of gastric cancer cells similar to that of the overexpression of miR-375. Besides, overexpression of JAK2 could promote the migration and invasion of gastric cancer cells. Thus, we assumed that JAK2 might counteract the inhibitory effect on the cells migration and invasion caused by miR-375. Given the critical role of miR-375 and JAK2 as master regulators in gastric cancer cells proliferation, migration, and invasion, both of them has therapeutic potential in gastric cancer treatment. Therefore, it remains to be investigated whether there is other targets participate in miR-375 mediated gastric metastasis.

Of particular interest, we further studied how miR-375 involved in the gastric carcinogenesis. Although there is an evident that DNA methylation and histone deacetylation are the possible mechanisms involved in the downregulation of miR-375 in gastric cancer [23]. The mechanisms underlying miR-375 dysregulation in gastric cancer metastasis are still poorly understood. There is possibility for a transcriptional blockage of miR-375 gene expression in this process. Here we show that the metastasis associated transcription factor Snail is a potential upstream regulator of miR-375 expression. Snail is a DNA-binding zinc finger protein and has been reported as transcriptional repressor [28]. Acumulating evidences show that Snail binds to E-boxes in the promoter of E-cadherin and represses its transcription to regulate tumor invasion development [29]. Moreover, overexpression of Snail in cancers was found to be associated with lymph node metastasis, tumor relapse and prognosis [30], [31], [32], [33], [34], [35]. In consistent with other reports, our study found that Snail mRNA is overexpressed in gastric cancer tissues when compared with their adjacent non-malignant tissues. As the promoter activity of miR-375 could be suppressed by Snail and overexpression of Snail could reduce miR-375 expression level significantly, we further found a distinct inverse correlation between the expression level of miR-375 and the level of Snail mRNA in gastric cancer samples. Snail is also found to be involved in the regulation of gastric cancer cells migration by targeting miR-375.

In conclusion, we have identified that miR-375 was aberrantly expressed in the gastric cancer tissues from metastasis-positive patients or gastric cancer cells with greater migration and invasion activities when compared with tissues from metastasis-free patients or non-invasive gastric epithelial cell line GES-1 respectively. Overexpression of miR-375 reduced gastric cancer cells migration and invasion activities at least partially by targeting JAK2. Moreover, Snail may be an upstream regulator of miR-375 which negatively regulates the expression of miR-375 and was involved in the regulation of gastric cancer cells migration by targeting miR-375. Together, our results provide an undescribed pathway, in which a transcription factor Snail regulates the expression of miR-375, which suppresses its direct target JAK2, leading to inhibit the migration and invasion of gastric cancer cells. Our data indicates that restoration of miR-375 or inhibition of Snail or JAK2 may be useful therapeutic strategies for gastric cancer treatment. It certainly will be very interesting to further explore the potential effectiveness of those molecules targeting miR-375, JAK2 or Snail in the treatment of gastric cancer. Another interesting topic for future research will be the identification of other possible targets of miR-375, and, more specifically, the possible involvement of JAK2 in gastric cancer metastasis.

## Supporting Information

Figure S1 **Ectopic expression of miR-375 suppresses the migration of MGC-803 cells.** MGC-803 cells transfected with miR-375 precursor (miR-375), negative control (Negative) or neither of the above (Mock) were subjected to Transwell migration assay (A) and scratch-wound healing analysis (C). (A) Representative fields of invasive cells on the underside of membrane which were fixed and stained with DAPI. (B) Total number of migrated cells on the underside of membrane was counted by IPP 6.0 software. (C) The cells migration to the wounded area was photographed by microscopy at 0 h, 12 h, 24 h and 36 h post-wounding. The dotted lines indicate the areas lacking cells. (D) The rate of migration was examined by measuring the distance of cells moved from the wound edge toward the center in 36 h after scratching. The data are presented as mean ± SE of at least three independent experiments. Bars, 50 µm. *P<0.05, **P<0.01.
(TIF)

Figure S2 **Overexpression of JAK2 reverses miR-375 induced inhibition of MGC-803 cells migration and invasion.** The cells transfected with the indicated vectors or oligonucleotides were subjected with Transwell migration (A) or Matrigel invasion assay (C). The rescue experiments for miR-375 overexpression were performed by ectopic expression of JAK2 without 3'-UTR in miR-375-treated cells. (A, C) Representative fields of the cells on the bottom chamber at 12 h post migration or invasion were shown. Scale bars, 50 µm. (B, D) The total number of migrated or invasive cells from nine randomly chosen fields was counted by IPP 6.0. *P<0.05, **P<0.01.
(TIF)

Figure S3 **JAK2 has no effect on miR-375 expression.** The AGS and MGC-803 cells were transfected with JAK2 overexpression vector or control vector and subjected to RT-PCR analysis for the expression level of miR-375. The level of RNA U6 was used as control.
(TIF)
