# Supplementary figures and images for: Snail-Regulated MiR-375 Inhibits Migration and Invasion of Gastric Cancer Cells by Targeting JAK2
Source: PLoS One. 2014 Jul 23;9(7):e99516. doi: 10.1371/journal.pone.0099516 (PMC4108470; doi:10.1371/journal.pone.0099516)

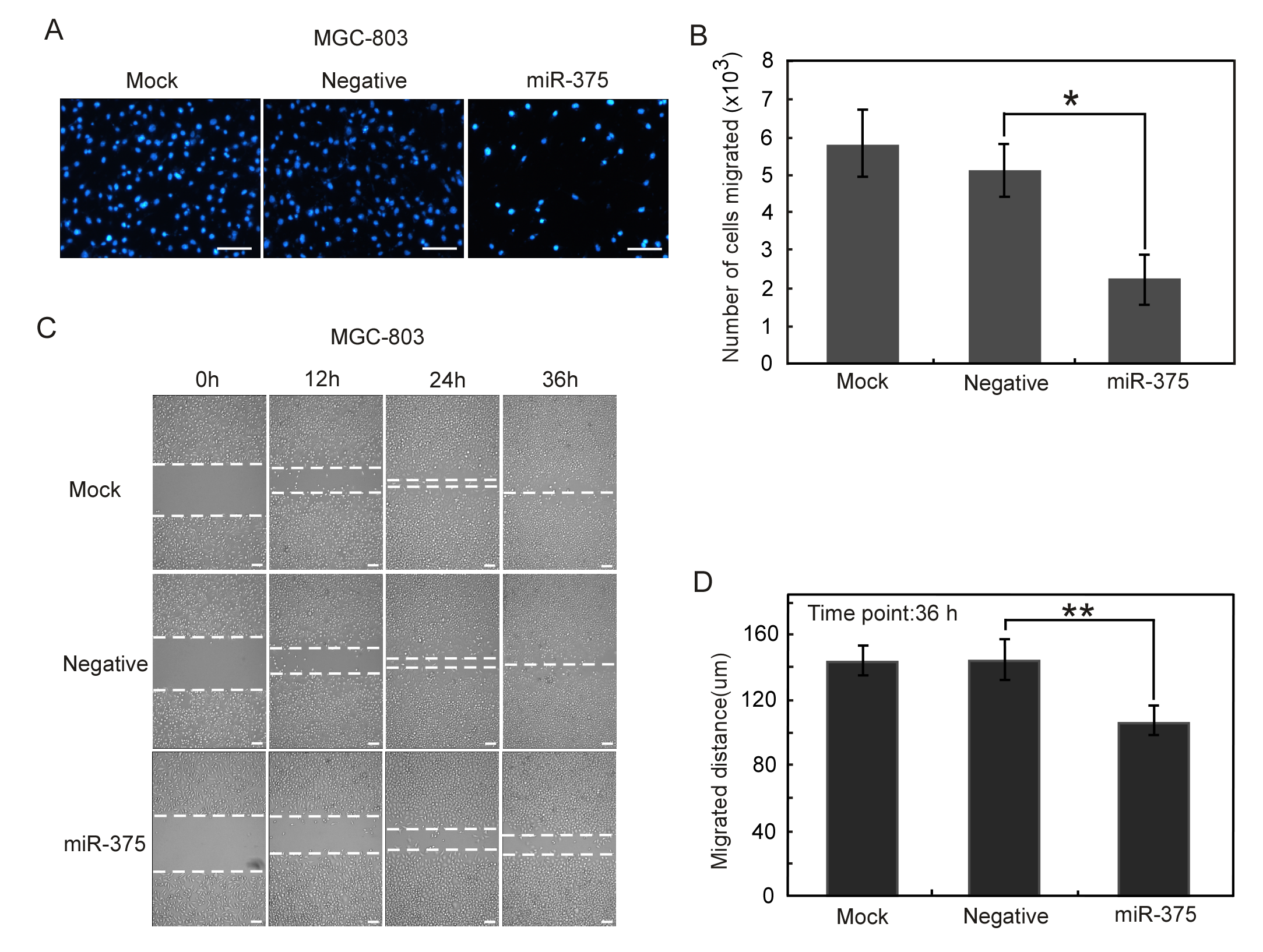

Supplement: Figure S1 — Ectopic expression of miR-375 suppresses the migration of MGC-803 cells. MGC-803 cells transfected with miR-375 precursor (miR-375), negative control (Negative) or neither of the above (Mock) were subjected to Transwell migration assay (A) and scratch-wound healing analysis (C). (A) Representative fields of invasive cells on the underside of membrane which were fixed and stained with DAPI. (B) Total number of migrated cells on the underside of membrane was counted by IPP 6.0 software. (C) The cells migration to the wounded area was photographed by microscopy at 0 h, 12 h, 24 h and 36 h post-wounding. The dotted lines indicate the areas lacking cells. (D) The rate of migration was examined by measuring the distance of cells moved from the wound edge toward the center in 36 h after scratching. The data are presented as mean ± SE of at least three independent experiments. Bars, 50 µm. *P<0.05, **P<0.01. (TIF) [file pone.0099516.s001.tif]

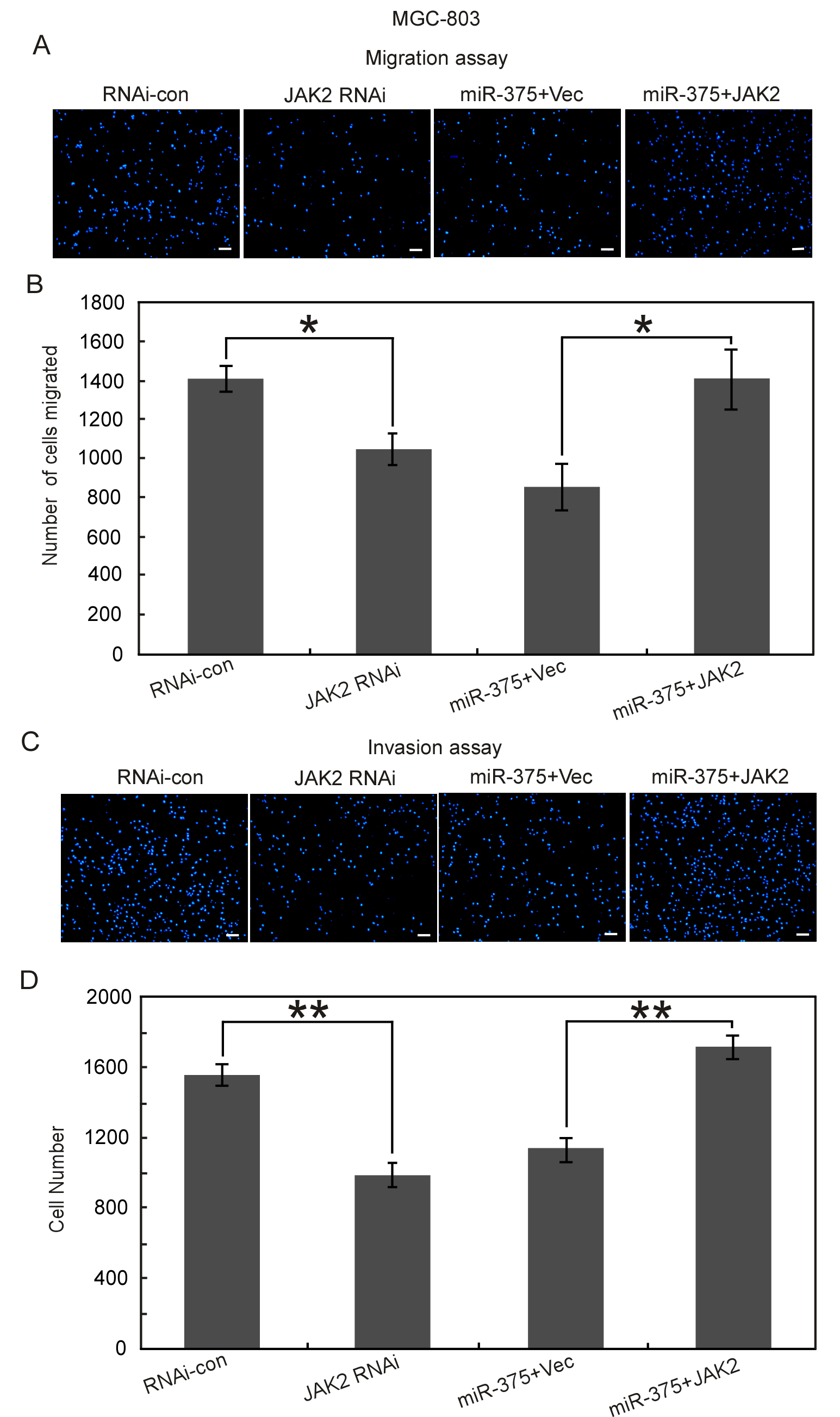

Supplement: Figure S2 — Overexpression of JAK2 reverses miR-375 induced inhibition of MGC-803 cells migration and invasion. The cells transfected with the indicated vectors or oligonucleotides were subjected with Transwell migration (A) or Matrigel invasion assay (C). The rescue experiments for miR-375 overexpression were performed by ectopic expression of JAK2 without 3'-UTR in miR-375-treated cells. (A, C) Representative fields of the cells on the bottom chamber at 12 h post migration or invasion were shown. Scale bars, 50 µm. (B, D) The total number of migrated or invasive cells from nine randomly chosen fields was counted by IPP 6.0. *P<0.05, **P<0.01. (TIF) [file pone.0099516.s002.tif]

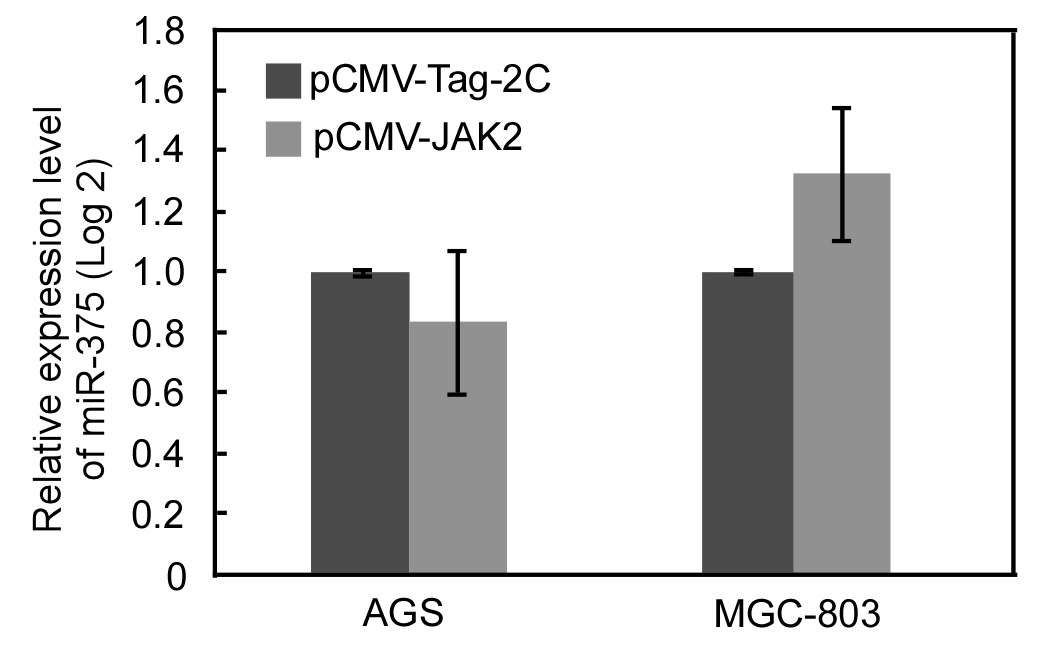

Supplement: Figure S3 — JAK2 has no effect on miR-375 expression. The AGS and MGC-803 cells were transfected with JAK2 overexpression vector or control vector and subjected to RT-PCR analysis for the expression level of miR-375. The level of RNA U6 was used as control. (TIF) [file pone.0099516.s003.tif]
